# Supplementary material for: Reconciling Mining with the Conservation of Cave Biodiversity: A Quantitative Baseline to Help Establish Conservation Priorities
Source: PLoS One. 2016 Dec 20;11(12):e0168348. doi: 10.1371/journal.pone.0168348 (PMC5173368; doi:10.1371/journal.pone.0168348)
Supplement: S1 Dataset — (ZIP) [file pone.0168348.s002.zip › Taxa/Serra Norte/SN_2013/N5S/Lista N5S-22.pdf]

CAVIDADE N5S-0022

Classe	Ordem	Fam/Outros	Gên/Outros	Espécie	S-U	U-S	Zon.
Arachnida	Acari	Prostigmata		jovem	X	X	
Arachnida	Amblypygi	Charinidae	*Charinus*	sp.1	1		P
Arachnida	Amblypygi	Phrynidae	*Heterophrynus*	*longicornis*	1	X	
Arachnida	Araneae	Araneidae	*Alpaida*	sp.1		X	
Arachnida	Araneae	Caponiidae		jovem	X		P/A
Arachnida	Araneae	Ctenidae		jovem		X	
Arachnida	Araneae	Filistatidae		sp.1	X		E
Arachnida	Araneae	Mimetidae	*Ero*	sp.1	X		P
Arachnida	Araneae	Ochyroceratidae		jovem	X	X	E/P/A
Arachnida	Araneae	Pholcidae		jovem	X		E
Arachnida	Araneae	Scytodidae	*Scytodes*	jovem	1		P
Arachnida	Araneae	Theridiosomatidae	*Plato*	sp.1	X	X	P
Arachnida	Palpigradi	Eukoeneniidae	*Eukoenenia*	sp.1		X	
Arachnida	Pseudoscorpiones	Bochicidae		sp.		X	
Arachnida	Pseudoscorpiones	Chthoniidae	*Pseudochthonius*	sp.1	X		P
Arachnida	Pseudoscorpiones	Chthoniidae	*Pseudochthonius*	sp.2		X	
Chilopoda	Geophilomorpha	Schendylidae		sp.1	2		P/A
Diplopoda	Polydesmida	Pyrgodesmidae		sp.1	X	X	A
Entognatha	Collembola	Paronellidae		sp.6	X		E
Insecta	Blattodea	Blattellidae		sp.5	1	X	E
Insecta	Coleoptera	Curculionidae	Platypodinae	sp.2	X		P
Insecta	Coleoptera	Curculionidae	Scolytinae	sp.1	X		P
Insecta	Coleoptera	Staphylinidae		sp.31	X		P
Insecta	Diptera	Calliphoridae		sp.	X		E/P
Insecta	Diptera	Chloropidae		sp.	X		P
Insecta	Diptera	Lauxaniidae		sp.	X		P
Insecta	Diptera	Micropezidae		sp.	X		P
Insecta	Diptera	Mycetophilidae		sp.		X	
Insecta	Diptera	Tephritidae		sp.	X		P
Insecta	Heteroptera	Dipsocoroidea		sp.	X	X	E/P
Insecta	Hymenoptera	Diapriidae		sp.3	X		P
Insecta	Hymenoptera	Formicidae	Myrmicinae	sp.1	X		E
Insecta	Hymenoptera	Scelionidae		sp.		X	
Insecta	Isoptera	Termitidae		sp.		X	
Insecta	Lepidoptera	Tineoidea		sp.2	X		E
Insecta	Neuroptera	Myrmeleontidae		jovem	X		E
Insecta	Odonata			sp.		X	
Insecta	Orthoptera	Phalangopsidae	*Phalangopsis*	sp.	15		P
Insecta	Psocoptera	Epipsocidae	*Epipsocus*	sp.2	X		P
Insecta	Thysanura	Nicoletiidae		jovem	X		E
Malacostraca	Isopoda	Scleropactidae	*Circoniscus*	*buckupi*	X		P/A
Mammalia	Chiroptera	Emballonuridae	*Peropteryx*	*kappleri*	3		P/A
